# Lithium Manganese Sulfates as a New Class of Supercapattery Materials at Elevated Temperatures

**DOI:** 10.3390/ma16134798

**Published:** 2023-07-03

**Authors:** Delyana Marinova, Mariya Kalapsazova, Zlatina Zlatanova, Liuda Mereacre, Ekaterina Zhecheva, Radostina Stoyanova

**Affiliations:** 1 Institute of General and Inorganic Chemistry, Bulgarian Academy of Sciences, 1113 Sofia, Bulgaria; maria_l_k@svr.igic.bas.bg (M.K.); zhecheva@svr.igic.bas.bg (E.Z.); radstoy@svr.igic.bas.bg (R.S.); 2 Faculty of Chemistry and Pharmacy, Sofia University “St. Kliment Ohridski”, 1164 Sofia, Bulgaria; nhzz@chem.uni-sofia.bg; 3 Institute for Applied Materials, Karlsruhe Institute of Technology, 76344 Eggenstein-Leopoldshafen, Germany; liuda.mereacre@kit.edu

**Keywords:** supercapattery, lithium-manganese sulfates, structural transformation, ionic liquid electrolyte, high temperature performance

## Abstract

To make supercapattery devices feasible, there is an urgent need to find electrode materials that exhibit a hybrid mechanism of energy storage. Herein, we provide a first report on the capability of lithium manganese sulfates to be used as supercapattery materials at elevated temperatures. Two compositions are studied: monoclinic Li_2_Mn(SO_4_)_2_ and orthorhombic Li_2_Mn_2_(SO_4_)_3_, which are prepared by a freeze-drying method followed by heat treatment at 500 °C. The electrochemical performance of sulfate electrodes is evaluated in lithium-ion cells using two types of electrolytes: conventional carbonate-based electrolytes and ionic liquid IL ones. The electrochemical measurements are carried out in the temperature range of 20–60 °C. The stability of sulfate electrodes after cycling is monitored by *in-situ* Raman spectroscopy and *ex-situ* XRD and TEM analysis. It is found that sulfate salts store Li^+^ by a hybrid mechanism that depends on the kind of electrolyte used and the recording temperature. Li_2_Mn(SO_4_)_2_ outperforms Li_2_Mn_2_(SO_4_)_3_ and displays excellent electrochemical properties at elevated temperatures: at 60 °C, the energy density reaches 280 Wh/kg at a power density of 11,000 W/kg. During cell cycling, there is a transformation of the Li-rich salt, Li_2_Mn(SO_4_)_2_, into a defective Li-poor one, Li_2_Mn_2_(SO_4_)_3_, which appears to be responsible for the improved storage properties. The data reveals that Li_2_Mn(SO_4_)_2_ is a prospective candidate for supercapacitor electrode materials at elevated temperatures.

## 1. Introduction

Nowadays, supercapattery systems emerge as promising energy storage technologies since they combine the high specific power of supercapacitors with the high energy density of rechargeable batteries into one device [1]. The function of supercapattery is based on hybrid capacitive and Faradaic charge storage reactions that rely on using capacitive materials as a negative electrode and battery-type or pseudocapacitive materials as a positive electrode [2]. As negative electrodes, activated carbons (ACs) or their composites are the materials of choice. In contrast to the negative electrodes, the selection of positive electrodes is still under intensive debate [2]. Till now, the most prospective positive electrodes are bi-metallic transition metal oxides, hydroxides, sulfides, and phosphates because they possess ions in multivalent oxidation states capable of Faradaic charge storage [2,3]. Comparing the anode and cathode materials, the bottleneck in the realization of a supercapattery device is the choice of the right cathode materials.

When searching for alternative positive electrodes, sulfate-based materials gain increasing attention due to their low ecological impact [4]. Recently, sulfate-based materials have become a new class of battery-like electrodes since they display a variety of tunnel-type structures and have redox potentials higher than those of conventional phosphate materials [4,5,6]. The main representatives of the sulfate electrodes are the double salts of Li, Na, and transition metal ions [6,7,8]. The sulfate electrodes store energy due to intercalation reactions of Li^+^ or Na^+^ into the tunnel structure of sulfates; charge compensation is achieved by transition metal ions through changes in their oxidation states [6,7,8]. Thus, selecting the structure type and nature of transition metal ions is important to designing battery-like sulfate electrodes. In the sodium family, several types of structures that are suitable for alkali intercalation have been identified depending on the ratio between sodium and transition metal ions: Na_2+2δ_Fe/Mn_2−δ_(SO_4_)_3_ with an alluaudite structure (SG *C*_2/*c*_) [7,8,9,10]; eldfellite NaFe(SO_4_)_2_ with a layered structure [11]; Na_2_Mn_3_(SO_4_)_4_ with an orthorhombic structure (SG *Cmc*21) [12]; Na_2_Fe(SO_4_)_2_ isostructural to α-Na_2_Co(SO4)_2_ [13]; kröhnkite-type structure Na_2_Fe(SO_4_)_2_·2H_2_O [14,15]. The best electrochemical performance is observed for alluaudite Na_2+2δ_Fe/Mn_2−δ_(SO_4_)_3_, whose potential of alkali intercalation increases after the replacement of Fe/Mn with cobalt or nickel ions [16,17,18]. Although Na_2_M_2_(SO_4_)_3_ adopts an alluaudite type of structure, the lithium analogues Li_2_M_2_(SO_4_)_3_ possess different crystal structures and electrochemical activities [19]. The iron and manganese salts Li_2_Fe_2_(SO_4_)_3_ and Li_2_Mn_2_(SO_4_)_3_ exhibit NASICON, anti-NASICON, and orthorhombic types of structures [20,21]. The iron analogues interact with Li^+^ at a potential of 3.6 V vs. Li^+^/Li [21,22], while the manganese analogue is electrochemically inactive in the voltage range of 3.0–4.3 V [23]. The inactive Li_2_Mn_2_(SO_4_)_3_ can be converted into an active intercalation matrix only when it is obtained from the interaction between Na_2_Mn_2_(SO_4_)_3_ alluaudite and lithium electrolyte [22]. Thus, the *in-situ* generated phase, Li_2_Mn_2_(SO4)_3_, intercalates at least 1 mol of Li in lithium-ion cells working with lithium electrolytes based on ionic liquid [22]. The highest potential of lithium intercalation is observed at lithium-rich sulfate salts, Li_2_Fe(SO_4_)_2_, having monoclinic and orthorhombic structures (i.e., the potential of ∼3.8 V vs. Li^+^/Li) [23,24,25], while the manganese analog Li_2_Mn(SO_4_)_2_ displays worse electrochemical behavior in lithium-ion cells [24].

Despite intensive studies on sulfate materials as battery-like electrodes, their capacitive properties remain unexplored. However, studies of the capacitive properties of polyanionic compounds (including mainly phosphates) have grown constantly during the last few years [26,27,28,29,30]. The advantages of using sulfate- and phosphate-based electrodes over oxide-based ones are their thermal stability in a charged state, which has an impact on the safety of energy storage devices at elevated temperatures [31,32,33,34].

The next important component in supercapattery systems is the electrolyte [35]. Given that the electrolyte provides redox ions for electrochemical reactions, state-of-the-art research has been devoted to finding electrolytes with more than one redox species so that they can achieve a high storage capacity [25,36]. Irrespective of the similar composition, the electrolyte has different electrochemical stability in batteries and supercapacitors due to the different energy storage mechanisms: for example, the conventional lithium carbonate-based electrolyte (i.e., 1 M LiPF_6_ in EC/EMC/DMC) is decomposed in lithium-ion cells and supercapacitors above 4.0 V and 3.0 V, respectively [37,38]. The replacement of carbonate-based electrolytes with ionic liquids (ILs) has been recognized to result in an improvement of the energy density of electrochemical devices and their safety since ILs exhibit better chemical and electrochemical stability [39,40,41,42]. Because of their high viscosity and low ionic conductivity, IL electrolytes are preferentially used at elevated temperatures, especially for rechargeable batteries [34,43,44]. Irrespective of the significance of IL electrolytes for battery and supercapacitor performance, little work has been devoted to the examination of supercapattery with IL electrolytes. Furthermore, most sulfate and phosphate electrodes are compatible with IL electrolytes, which is a precondition for increasing the energy density of supercapattery devices.

This study provides a first report on the capability of lithium manganese sulfates to be used as supercapattery materials at elevated temperatures. Two compositions are studied: monoclinic Li_2_Mn(SO_4_)_2_ and orthorhombic Li_2_Mn_2_(SO_4_)_3_. For the sulfate preparation, a freeze-drying method is adopted. This method has been previously shown to be suitable for the formation of Li_2_Mn_2_(SO_4_)_3_ [22]. The electrochemical performance of sulfate electrodes is evaluated in lithium-ion cells using two types of electrolytes: conventional carbonate-based electrolytes and IL ones. The electrochemical measurements are carried out in the temperature range of 20–60 °C. The stability of sulfate electrodes after cycling is monitored by *in-situ* Raman spectroscopy and *ex-situ* XRD and TEM analysis. The data of this study enables us to highlight two novel aspects of sulfate electrode materials: on the one hand, we demonstrate a hybrid mechanism of charge storage by lithium manganese sulfates, and on the other, we reveal the impact of the electrolytes and elevated temperatures on the Li-storage mechanism.

## 2. Results and Discussion

### 2.1. Structural Characterization of Li_2_Mn(SO_4_)_2_ and Li_2_Mn_2_(SO_4_)_3_

**Figure 1:**
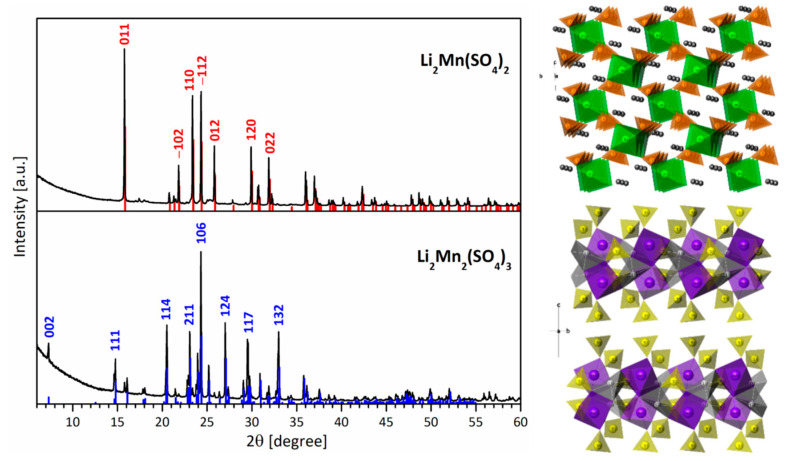
Experimental XRD patterns (**left**) and schematic view of crystal structure (**right**) for L2M and L2M2. The indexation of XRD patterns of both salts is indicated with | and | lines, respectively. In addition, the crystal planes corresponding to the intensive diffraction peaks are also given.

Freeze-drying of aqueous solutions of lithium and manganese sulfates yields a phase mixture between Li_2_SO_4_ and MnSO_4_. After thermal treatment at 500 °C, mixed Li_2_SO_4_ and MnSO_4_ salts (denoted as precursors) are transformed into double salts of lithium and manganese (Figure 1). Depending on the Li-to-Mn ratio in the precursors, the XRD patterns are indexed to Li_2_Mn(SO_4_)_2_ and Li_2_Mn_2_(SO_4_)_3_ phases, respectively. For the sake of convenience, two compositions, Li_2_Mn(SO4)_2_ and Li_2_Mn_2_(SO4)_3_, will be denoted by L2M and L2M2, respectively. Although L2M adopts a monoclinic crystal structure, L2M2 crystallizes in an orthorhombic structure (Figure 1). The lattice parameters of the L2M and L2M2 phases are listed in Table 1, and they coincide with those previously reported [21,45]. The structure of L2M can be described as a tunnel-type one, which is isostructural to iron and cobalt analogues [46]. The structure consists of alternating vertex-bonded MnO_6_ octahedra and SO_4_ tetrahedra, sharing between them common oxygen atoms, thus forming chains [45]. The lithium atoms are isolated and located in the channels between the chains. In contrast to L2M, the structure of L2M2 forms a 3D grid built by SO_4_, LiO_4_, and MnO_6_ polyhedra [21]. Every two manganese polyhedra are edge-shared and connected via three bridging sulfate tetrahedra, forming the well-known Sc_2_(WO_4_)_3_-type structure [47]. The last half of the MnO_6_ polyhedra are distributed independently in the structure. As in the case of MnO_6_ polyhedra, half of LiO_4_ polyhedra are involved in edge-shared Li_2_O_6_ bitetrahedra, whereas the other half are independently distributed.

**Figure 2:**
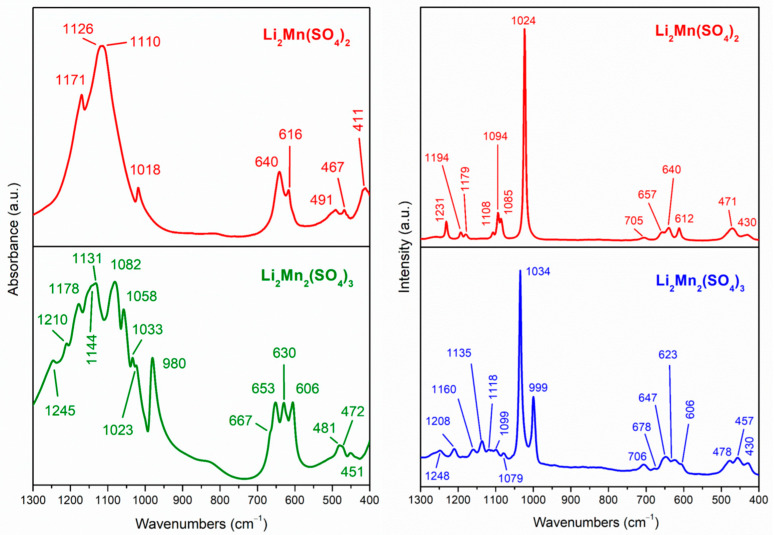
IR and Raman spectra of L2M and L2M2 in the region of normal vibration of the sulfate ions.

IR spectroscopy complemented by Raman scattering provides further characteristics on the structural peculiarities of L2M and L2M2 (Figure 2). In the region 1200–400 cm^−1^, the IR and Raman spectra are dominated by the vibration modes of SO_4_ groups, in accordance with our previous studies on kröhnkite, blödite, and alluaudite sulfate salts [10,48]. Between 900 and 1200 cm^−1^, the vibration spectra of SO_4_ groups display characteristic bands due to asymmetric and symmetric stretching modes (ν_3_ and ν_1_), while between 500–600 and 400–500 cm^−1^, the asymmetric and symmetric bending modes (ν_4_ and ν_2_) appear [49].

The assigned modes of SO_4_ groups for L2M and L2M2 are listed in Table 2. The comparison reveals quite different vibration spectra for SO_4_ groups in L2M2 and L2M salts. This is a consequence of their different crystal structures, where an SO_4_ group occupies one crystallographic position for L2M and three non-equivalent sites for L2M2. Thus, only one ν_1_ mode is clearly resolved for L2M, while at least three ν_1_ modes occur for L2M2. In addition to this, the ν_3_-modes are spread over a broader range for L2M2 than that for L2M (i.e., between 1180 and 1060 cm^−1^ for L2M2 versus 1170 and 1110 cm^−1^ for L2M). In accordance with ν_1_ and ν_3_ modes, the ν_4_ and ν_2_ modes of SO_4_ are split into more components for L2M2 salts than for L2M. All these vibrational findings corroborate the crystal structures adopted by L2M and L2M2.

**Figure 3:**
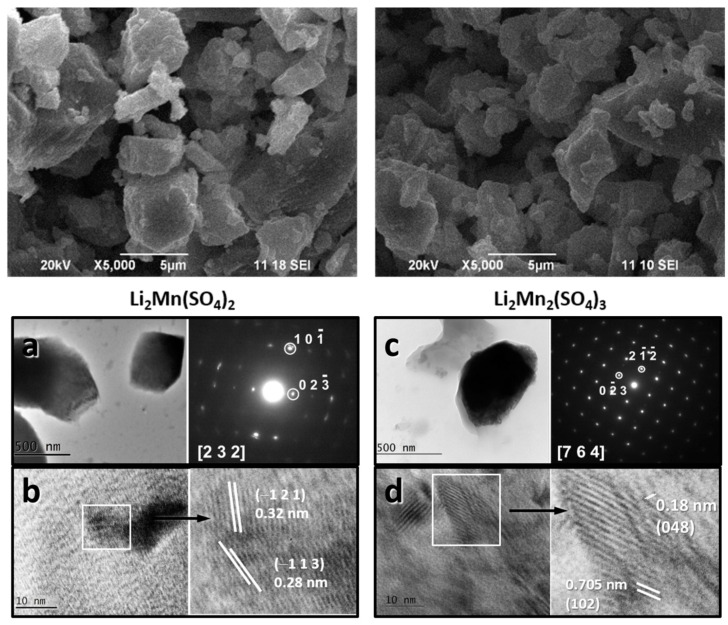
SEM images of L2M (**a**) and L2M2 (**b**). TEM bright field micrographs, SAED, and HR-TEM of L2M (**c**) and L2M2 (**d**).

Both L2M and L2M2 exhibit similar morphology, which consists of micrometric plate-like aggregates (Figure 3). Inside aggregates, well-crystallized particles with dimensions of about 500–700 nm are clearly resolved. SAED and HR-TEM confirm the formation of distinct L2M and L2M2 phases in L2M and L2M2 compositions, respectively.

### 2.2. Lithium Storage by L2M and L2M2

**Figure 4:**
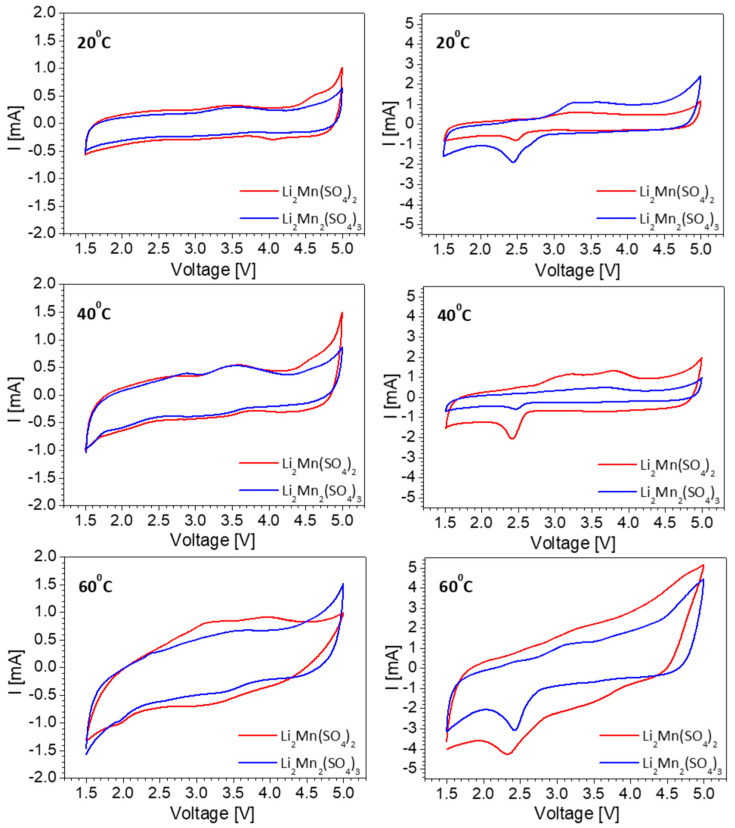
CV curves of L2M (red lines) and L2M2 (blue lines) at 20, 40, and 60 °C in electrolytes of 1 M LiPF_6_ in EC/DMC (**left**) and LiTFSI-Pyr_1,3_FSI (**right**). The scan rate is 50 mV/s.

To understand the lithium storage properties, Figure 4 compares the CV curves of L2M and L2M2 in lithium-ion cells using two kinds of lithium electrolytes: the conventional carbonate-based electrolyte and IL electrolytes. The electrochemical reaction proceeds at a broad potential range (i.e., between 1.5 and 5.0 V) at room temperature and at elevated temperatures. As one can see, the CV curves for L2M and L2M2 electrodes are similar. In a carbonate-based electrolyte, the CV curves display capacitive profiles with barely resolved Faradaic peaks between 2.0 and 4.0 V. When the carbonate-based electrolyte is replaced with IL electrolyte, the Faradaic peaks become more clearly visible. The role of the Faradaic reactions also increases upon raising the recording temperature from 20 to 60 °C. These results disclose that Li storage by L2M and L2M2 takes place by a hybrid mechanism that depends on the kind of electrolyte used and the recording temperature.

**Figure 5:**
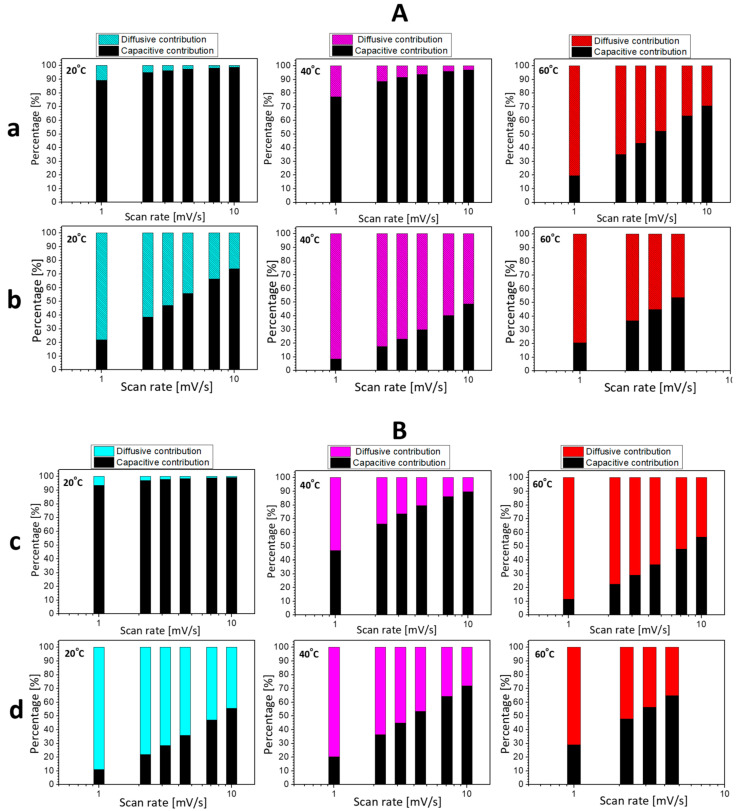
The contribution of capacitive and Faradaic (i.e., diffusive) reactions to the storage of Li by L2M (**A**) and L2M2 (**B**) in carbonate-based (**a**,**c**) and IL (**b**,**d**) electrolytes at 20 °C, 40 °C, and 60 °C.

To quantify the contribution of capacitive and Faradaic reactions towards Li-storage, the CV curves are analyzed taking into account the dependence of the current (i) on the scan rate (v) following the equation: i(v) = k_1_v + k_2_v^1/2^ [50]. The results are summarized in Figure 5, so that several features for Li-storage by L2M and L2M2 can be identified. The electrochemical mechanism of Li-storage looks similar for both L2M and L2M2 salts. In carbonate-based electrolytes, the storage reactions are dominated by capacitive ones irrespective of the scan rate (i.e., more than 90%), while in IL electrolytes, the contribution of Faradaic reactions is more significant (i.e., between 20 and 80%). The elevated temperatures favor the proceeding of Faradaic reactions at the expense of the capacitive ones, especially when the electrochemical reaction takes place in a carbonate-based electrolyte enhancing the temperature from 20 to 40 and 60 °C, the contribution of the Faradaic reaction increases smoothly from about 0% to 5% and 30% for L2M and from 0% to 15% and 40% for L2M2, respectively. In comparison with carbonate-based electrolytes, in IL electrolytes, the elevated temperatures slightly affect the mechanism of the electrochemical reaction; the contribution of the Faradaic reaction varies between 20% and 80% depending on the scan rate.

**Figure 6:**
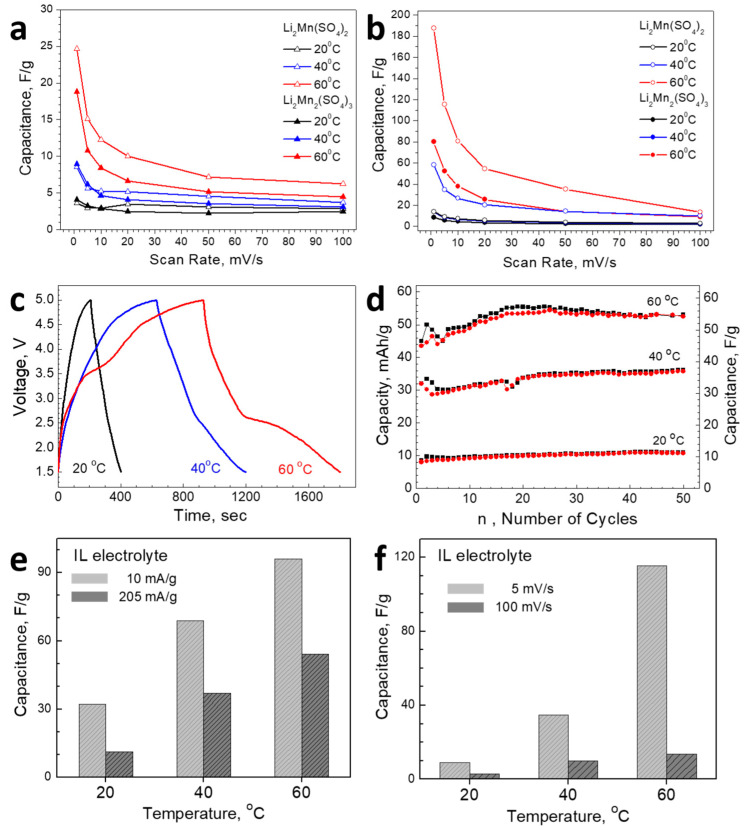
Capacitance (calculated from CV curves) delivered by L2M and L2M2 in carbonate-based (**a**) and IL (**b**) electrolytes. Charge-discharge curves of L2M cycled in IL electrolytes at 20, 40, and 60 °C (**c**). Cycling stability of L2M electrodes cycled in IL electrolytes at 20, 40, and 60 °C (**d**). In addition to the capacity (left Y scale), the corresponding capacitance is also given (right Y scale). The capacitance of L2M is determined from GCD at a current load of 10 and 205 mA/g as a function of operating temperature (**e**). The capacitance of L2M, determined from CV at a scan rate of 5 and 100 mV/s as a function of operating temperature (**f**). The IL electrolyte is used for (**e**,**f**) experiments.

Given the electrolyte- and temperature-induced changes in the Li-storage mechanism, one could expect L2M and L2M2 to exhibit different electrochemical performances in two types of electrolytes as well as at elevated temperatures. The evaluation of the electrochemical performance of sulfate salts is based on the calculated capacitance from CV curves using a scan rate between 1 and 100 mV/s. The calculated capacitances of L2M and L2M2, depending on the kind of electrolyte used and recording temperature, are shown in Figure 6. The comparison shows that, at room temperature, L2M and L2M2 display similar performance, where the capacitance reactions dominate the Li-storage mechanism: about 10 F/g at 25 °C in carbonate-based and IL electrolytes (Figure 6a,b). Contrary, at elevated temperatures, L2M outperforms L2M2 in respect of the magnitude of capacitance: at 60 °C and a slow scan rate, the capacitance for L2M reaches in carbonate-based electrolyte a value of 25 vs. 18 F/g for L2M2, as well as in IL electrolyte a capacitance of 180 F/g versus 85 F/g, respectively. This reveals an enormous increase in the capacitance of sulfate salts after the enhancement of the recording temperature. At these conditions, the contributions of the Faradaic reactions reach about 30–40% for L2M and L2M2. It is important that both L2M and L2M2 sulfates deliver a higher capacitance in the IL electrolyte, where the contribution of the Faradaic reactions is more significant. In addition, after the increase in the contribution of the Faradaic reactions, the dependence of the capacitance on the scan rate becomes stronger.

Based on CV experiments, it appears that the best performance is achieved for the L2M electrode when it cycles at elevated temperatures in the IL electrolyte. The better performance of L2M is further analyzed by the galvanostatic charge/discharge technique. Figure 6c compares the charge/discharge profiles for L2M cycled in IL electrolyte at 20, 40, and 60 °C. The comparison shows that charge/discharge curves contain humps, which become clearly visible at 60 °C. This gives evidence for the occurrence of Faradaic reactions in addition to capacitive ones, which is in accordance with CV experiments (Figure 5). Figure 6d gives the cycling stability of L2M as a function of operating temperature. Because of the hybrid mechanism of charge storage, the capacity of L2M at a current load of 205 mA/g and the corresponding capacitance are depicted in Figure 6. As one can see, the capacity increases slightly up to the 25th cycle, where the almost constant value is reached and the Coulombic efficiency tends to be around 99%. As in the case of the CV experiments, the capacity of L2M increases with the enhancement of the operating temperature from 20 to 60 °C. For the sake of better comparison, Figure 6e,f show a cross section at 20, 40, and 60 °C of the capacitance determined from GCD and CV experiments at a current load of 10 and 205 mA/g and scan rates of 5 and 100 mV/s, respectively. The comparison clearly supports the improved performance of L2M at elevated temperatures.

**Figure 7:**
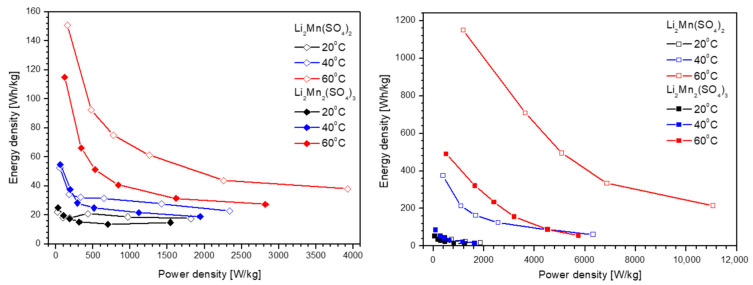
The Ragone plot for lithium-ion cells with L2M and L2M2 electrodes working in carbonate-based (**left**) and IL (**right**) electrolytes at room and elevated temperatures.

The electrochemical properties of lithium-ion cells using L2M and L2M2 electrodes allow us to evaluate the relationships between their energy density and power density (Figure 7). At room temperatures, cells with L2M and L2M2 display similar energy densities, which slightly decrease with increasing power densities (i.e., the energy density of around 20 Wh/kg at a power density varying between 50 and 1800 W/kg for L2M and L2M2). By enhancing the recording temperature, the energy density increases dramatically across the whole range of power densities. The best performance is obeyed by the cell with L2M electrode working in IL electrolyte at 60 °C: the energy density is around 1150 Wh/kg at a power density of 1200 W/kg, which is much higher than that with L2M2 electrode (i.e., 400 Wh/kg at 1200 W/kg). Even in the case of higher power energy (i.e., at 6000 W/kg), the energy density for the cell with L2M is still higher than that of the L2M2 analogue (i.e., 450 Wh/kg vs. 100 Wh/kg, respectively). It is worth mentioning that the L2M-based cell exhibits an energy density of 280 Wh/kg at a power density of 11,000 W/kg at 60 °C, these values being among the highest reported previously [27,51,52]. In comparison, the mixed nickel-manganese oxides with the ilmenite-type structure (i.e., NiMnO_3_) are among the best-performing pseudocapacitance electrodes, with an energy density of 65 Wh/kg at a power density of 3200 W/kg at 20 °C [27]. For example, the battery-like device composed of NiMnO_3_ ilmenite as a positive electrode and activated carbon as a negative electrode displays a maximum energy density of 132 Wh/kg at a power density of 1651 W/kg [52]. In addition, a supercapattery device of the type 3D-C-NiCo_2_O_4_/Ni||3D-Fe_3_S_4_@NiCo/SS achieves a maximum energy density of 49.8 Wh/kg and a power density of 8100 W/kg [53].

For the supercapattery device based on Ni_0.75_Mn_0.25_(PO_4_)_2_ positive electrode, it has been reported that it has an outstanding specific energy of 64.2 Wh/kg with a maximum specific power of 11,896 W/kg at 20 °C [26]. All these results reveal that L2M is a prospective candidate for supercapattery electrode materials at elevated temperatures.

### 2.3. Sulfate Transformation during Cycling

**Figure 8:**
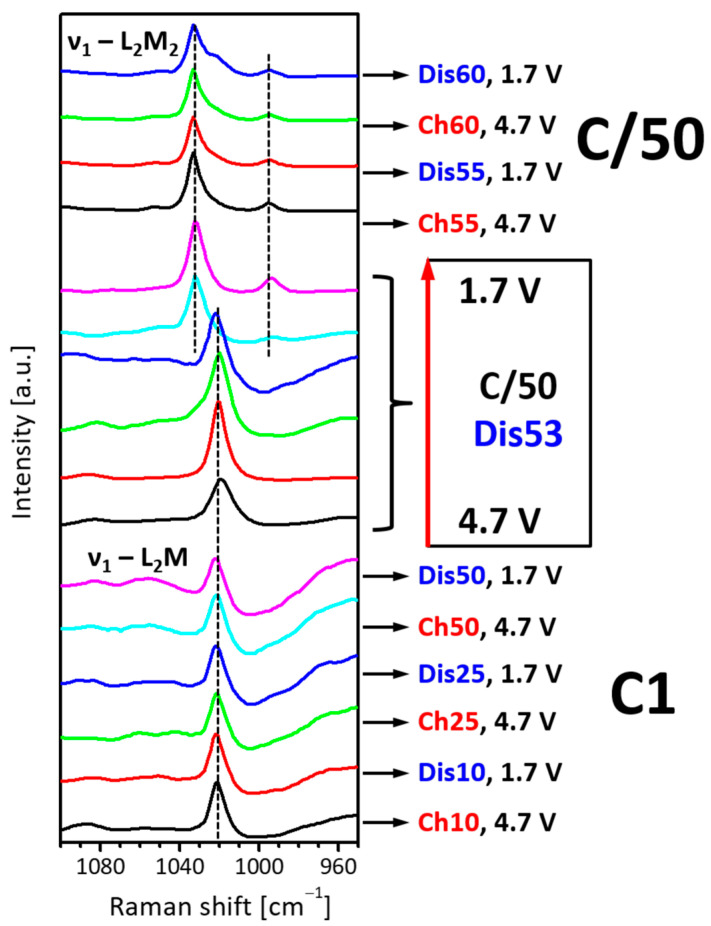
*In-situ* Raman spectra of L2M electrode cycled in a lithium-ion cell at 20 °C between 1.5 V and 5.0 V with a charging rate of C/1 (the first 50 cycles), followed by a charging rate of C/50 (the next 10 cycles). A carbonate-based electrolyte is used.

To rationalize the good electrochemical performance of L2M, *in-situ* Raman spectroscopy is undertaken. Because of the sensitivity of the ν_1_ mode of SO_4_ groups, the Raman spectra of L2M electrodes cycled between 5.0 V and 1.5 V in a carbonate-based electrolyte are shown in a wavelength range of 1100 and 950 cm^−1^ (Figure 8). Starting with a fast charging rate (i.e., a current load of 205 mA/g), the Raman spectra of charged and discharge electrodes show one band at 1024 cm^−1^ due to ν_1_-vibration of the SO_4_-group in the L2M structure. After 50 cycles, the current load is decreased from 205 to 4 mA/g, and the cell is cycled for the next 10 cycles while preserving the voltage range. At 53 cycles of discharging, the Raman is changed: the band at 1024 cm^−1^ is shifted to 1034 cm^−1^ concomitant with the appearance of a band at 995 cm^−1^. These SO_4_-modes correspond to the ν_1_ vibrations for L2M2, as shown in Figure 2. During cycling, another component appears as a shoulder at 1022 cm^−1^ (i.e., after 55 cycles of discharging). The next cycles do not provoke any further changes in the Raman spectra. This is an indication of a structural transformation of L2M into L2M2 during cycling. The *in-situ*-generated L2M2 is, most probably, responsible for the electrochemical performance.

**Figure 9:**
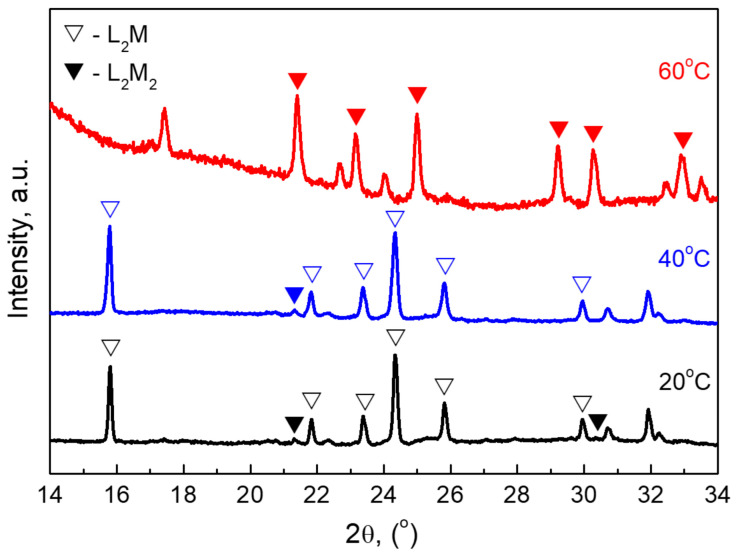
*Ex-situ* XRD patterns of L2M electrodes cycled in a lithium-ion cell at 20, 40, and 60 °C in IL electrolyte.

Furthermore, the phase transformation in L2M after cycling is monitored by *ex-situ* X-ray diffraction. Figure 9 collects the XRD patterns of L2M electrodes after 50 charge/discharge cycles at 20, 40, and 60 °C in IL electrolytes. The phase analysis of *ex-situ* XRD patterns reveals that L2M electrodes represent a mixture between L2M and L2M2 phases, whose ratio depends on the operating temperature: 80-to-20, 79-to-21, and 5-to-95% for the electrode cycled at 20, 40, and 60 °C, respectively. This means that at 60 °C, there is a nearly full phase transformation from L2M to L2M2 after cycling. This is an operating temperature where the L2M electrode performs best.

**Figure 10:**
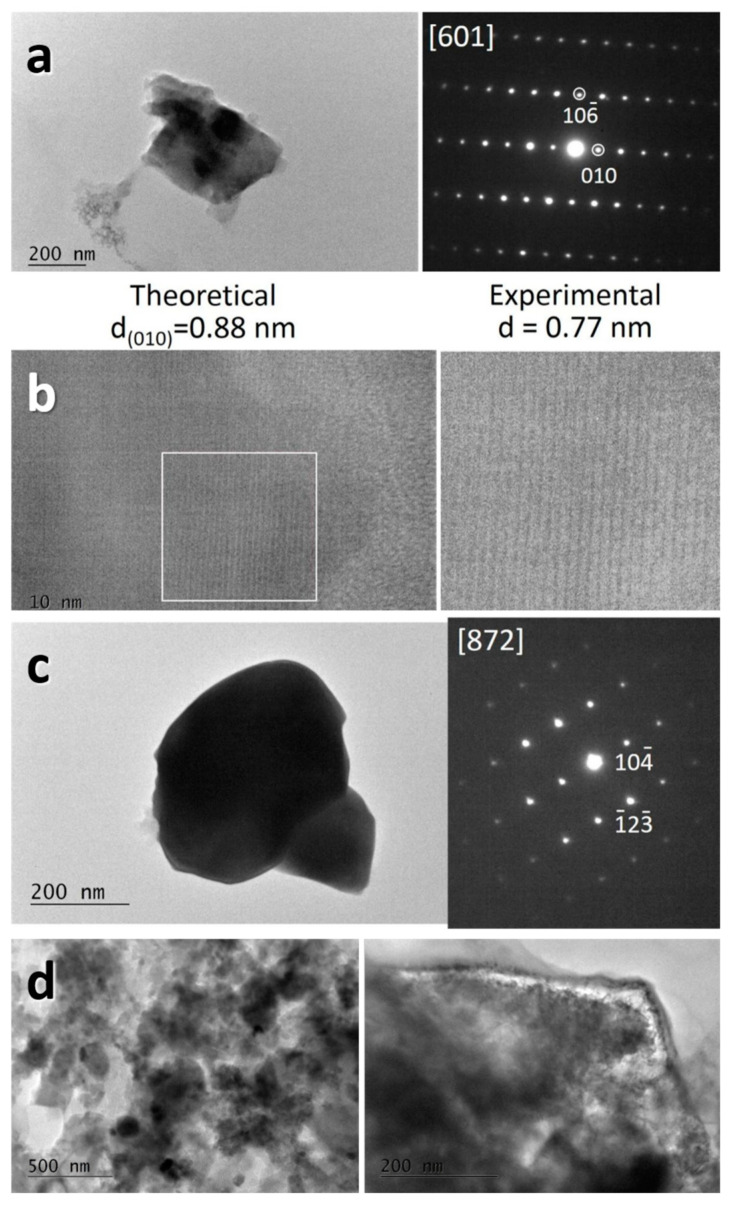
Bright-field images and SAED of L2M cycled in a lithium-ion cell with carbonate-based electrolyte at 20 °C; the indexation corresponds to the L2M2 phase (**a**). HR-TEM images of the (010) plane for *in-situ* generated L2M2 and its changes under the electron beam (**b**). In bright-field images and SAED of L2M2 cycled in a lithium-ion cell with a carbonate-based electrolyte at 20 °C, the indexation corresponds to L2M2 phase (**c**). Bright-field images of L2M cycled in a lithium-ion cell at 60 °C (**d**).

The change in L2M phase during cycling is also examined by *ex-situ* TEM analysis (Figure 10). The bright-field images demonstrate that the morphology of L2M and L2M2 remains intact during cycling. The SAED of the L2M electrode gives evidence for the formation of well-crystallized particles with a composition of L2M2, thus corroborating the Raman finding for the structural transformation of L2M into L2M2 during cycling. Furthermore, when we try to catch the HR-TEM image, the lattice fringes of the (001)-plane decrease constantly from 0.88 nm to a stable value of 0.77 nm. This is TEM evidence for the formation of the defect phase of L2M2, which undergoes shrinkage under the electron beam. In comparison with L2M, the L2M2 phase retains its composition during cycling. In addition, the L2M2 phase worked in a carbonate-based electrolyte is stable under an electron beam in contrast to the *in-situ* generated L2M2. Thus, TEM observations allow us to distinguish the *in-situ* generated L2M2 phase from the prepared in advance L2M2 phase, as well as explain their different electrochemical responses in lithium-ion cells.

After cycling at 60 °C, L2M preserves its morphology as a whole (Figure 10d). When we try to take a high-resolution image, it seems that L2M is decomposed under an electron beam. This implies that electrochemically generated L2M2 at 60 °C is structurally instable. On the one hand, this implies the formation of the defect-richer phase, and on the other hand, the defect density is a function of the operation temperature. The higher the operating temperature, the more defective phases are formed.

The phase transformation during cycling has already been established for hybrid metal-ion batteries [54]. In Li,Na and K,Na cells, which operate by Faradaic reactions, the dual storage of alkali ions proceeds in oxide-based electrodes by phase separation into nano-domain scales. The unique mechanism of dual alkali ion storage leads to a significant improvement in both rate capability and cycling stability [54,55,56]. Because of the hybrid mechanism of charge storage, the electrode design (i.e., structure and morphology design) plays a key role in the control of supercapattery performance. In this respect, the coating of pseudo-capacitive oxide, MnO_2−x_ containing oxygen vacancies, by CoS has been shown to yield a core-shell highly conductive structure, which exhibits in a supercapattery device with an aqueous electrolyte a high energy density of 34.72 Wh/kg at 597.24 W/ kg [57]. In addition to the coating technique, the surface modification (such as deposition of Co(OH)_2_ on the well-known cobalt phosphate) has a positive impact on the supercapattery performance [58]. Another approach includes the formation of composites between cobalt sulfide and cobalt phosphate, where a synergistic effect achieves a high energy density of 34.68 Wh/kg at a remarkable power of 13,600 W/kg [59]. Although many studies have been devoted to the study of the performance of electrodes at room temperature, little work is carried out at elevated temperatures. An excellent performance in the temperature range of 35–41 °C has been found for cobalt phosphate nano-/microstructure [60]. In this study, we demonstrated that the structural transformation of L2M into L2M2 is responsible for improving storage performance, especially at 60 °C.

## 3. Materials and Methods

### 3.1. Synthesis Procedure

Lithium-manganese sulfates with compositions L2M and L2M2 were prepared by the same method, which consists of freeze-drying reactions and heat treatment at 500 °C. This method has already been elaborated by us for the preparation of the L2M2 phase [22]. The aqueous solutions of lithium and manganese sulfates in a ratio Li:Mn = 2:1 and 1:1 were frozen immediately and dried at a temperature of −100 °C and pressure of up to 0.05 mBar in a time frame of 24 h by using the freeze dryer CHRIST (model Alpha 3-4 LSC basic apparatus, Martin Christ Gefriertrocknungsanlagen GmbH, Osterode am Harz, Germany). Thus, obtained fluffy white powders were annealed at 500 °C in an argon atmosphere for 72 h. This method yields L2M and L2M2.

### 3.2. Characterization Methods

The diffractometer Bruker D8 Advance (Bruker Corporation, Karlsruhe, Germany) with Cu Kα radiation was used for collecting the X-ray powder diffraction patterns within the range from 6 to 60 2θ with a step of 0.02 2θ and a counting time of 04 s/strip. The powder pattern program for calculating the experimental diffractograms was PowderCell 2.4.

The scanning electron microscope JEOL JSM 6390 (JEOL Ltd., Tokyo, Japan), equipped with an Oxford INCA energy dispersive X-ray spectrometer, was a monitoring instrument for the morphology and chemical composition of the lithium-manganese sulfates.

TEM investigations were performed on a JEOL 2100 transmission electron microscope (JEOL Ltd., Tokyo, Japan) in combination with a JEOL 2100 XEDS from Oxford Instruments at 200 kV. The specimens were milled in an agate mortar in ethanol and dispersed by ultrasonic treatment for several minutes. A droplet of the suspension was then dripped on standard holey carbon/Cu grids.

The infrared spectra were recorded on an FT interferometer (Thermo Scientific, Waltham, MA, USA) model Nicolet iS5 (resolution < 2 cm^−1^) at ambient temperatures using KBr discs as matrices. No ion exchange or other reactions with KBr have been observed during the preparation or IR measurements (i.e., the color of the pellets remains transparent). The Raman spectra were recorded with a Raman microscope (HORIBA, Ltd., Kyoto, Japan) of type HORIBA LabRam Evolution HR (Laser wavelengths: HeNe-Laser (633 nm, 17 mW)). The laser power on the sample was kept below 0.1 mW so that no heating effects on the powder sample could be observed. The nominal excitation power at the sample was ~0.35 mW to 8 mW, which was attenuated using a 10% filter. The excitation light was focused and collected using a ×50 LWD objective lens, resulting in a spot size on the sample surface was ~1.9 μm. The scattered light was dispersed using a 600 grooves/mm grating.

### 3.3. Electrochemical Characterization

The electrochemical cycling voltammetry curves of lithium manganese sulfates were examined using Swagelok-type three-electrode cells, while two electrode cells were utilized for the galvanostatic charge/discharge experiments. The cells are composed of Li metal as the negative (and reference) electrode, L2M or L2M2 as the positive electrode, and 1 M LiPF_6_(EC:DMC) or LiTFSI-Pyr_1,3_FSI (1:9) as the electrolytes. The positive electrode was a mixture containing 60% active lithium manganese sulfates, 30% Super C65 (TIMCAL), and 10% polyvinylidene fluoride (PVDF). The amount of carbon additives C65 and PVDF binder is chosen to improve the electrical conductivity of sulfate salts. The slurry was cast on carbon-coated aluminum foil and, after that, dried at 80 °C overnight. The disk electrodes with a diameter of 10 mm were cut, pressed, and dried at 80 °C under vacuum. The loaded mass of active materials on Al collectors was about 3–4 mg. The electrolyte was a 1 M LiPF_6_ solution in ethylene carbonate and dimethyl carbonate (1:1 by volume) with less than 20 ppm of water and LiTFSI-Pyr_1,3_FSI (Lithium bis(trifluoromethanesulfonyl)imide in N-methyl, propyl pyrrolidinium bis(fluorosulfonyl)imide) 1:9 by ratio). The lithium negative electrodes consisted of a clean lithium metal disk with a diameter of 9 mm. The cells were assembled in a glovebox (MB-Unilab Pro SP (1500/780), content of H_2_O and O_2_ under 0.1 ppm) under an argon atmosphere. The electrochemical reactions were carried out using a Biologic VMP-3e multi-channel potentiostat/galvanostat and impedance meter in potentiostatic mode. The model lithium half-cell was cycled in a window from 5.0 to 1.5 V at scan rates between 100 mV/s and 1 mV/s. Electrochemical tests at high temperatures were performed using thermal chambers with compressor cooling (Binder KB 53). The electrochemical cells for *in-situ* Raman spectroscopy are coin cell type 2032 with a glass window. The cells were mounted inside a glove box with electrolyte LP30 and a mass loading of 6–8 mg active material. Battery performance was tested on a BioLogic VSP300 battery tester at room temperature. For *ex-situ* TEM experiments, the electrochemical cell after 120 cycles and a switch of 1.5 V was dismounted inside the glove box, and the electrode was washed with DMC. This electrode is further analyzed by TEM.

For the calculation of the capacitance (F/g) from the cycling voltammetry curves, the following equations were used [27]:

C = C_el_/(m × ∆V), (1)

where C_el_ (coulomb) is electric charge for one cycle, ∆V (voltage) is voltage window, and m (grams) is mass of the active material. The capacity (mAh/g) is calculated following Q = (I × ∆t)/(m × 3.6).

The Energy densities (E, Wh/kg) and Power densities (P, W/kg) were calculated on the base of data for capacitance as [27]:

E = ½C × ∆V^2^, (2)

P = E × 3600/t(3)

where t(s) is the discharge time.

## 4. Conclusions

The freeze-drying method, followed by heat treatment at 500 °C, is an effective synthetic procedure for the preparation of L2M and L2M2 phases belonging to the family of double sulfate salts of lithium and manganese. They adopt monoclinic and orthorhombic structures, in which Li^+^ and Mn^2+^ ions are tetrahedrally and octahedrally coordinated. The SO_4_ groups occupy one crystallographic position for L2M, while three non-equivalent sites are observed for L2M2. Because of the application of one and the same synthetic method, the morphology of two distinct phases is similar and consists of micrometric aggregates composed of well-crystallized particles with dimensions varying between 500 and 700 nm.

Because of their structure and availability of Mn^2+^ ions, the double sulfate salts serve as electrodes in non-aqueous Li-ion cells. They store Li^+^ reversibly by a hybrid mechanism including capacitive and Faradaic reactions. In carbonate-based electrolytes, the capacitive reactions prevail, while in IL-based electrolytes, the role of the Faradaic reactions increases. The elevated temperatures favor the progression of Faradaic reactions when carbonate-based electrolytes are used, while a non-significant effect is observed in IL-based electrolytes.

Irrespective of the kind of electrolyte used and the recording temperature, the Li-rich salt, L2M, outperforms the Li-poor one, L2M2. This is a consequence of the transformation of the Li-rich salt, L2M, into a defective Li-poor one, L2M2. It appears that the *in-situ* generated Li-poor salt has better performance than the Li-poor one prepared prior to the electrochemical experiment. The capacitance of L2M increases with operating temperature, reaching values of 96 F/g and 54 F/g at a current load of 10 and 205 mA/g. At room temperature, the cell based on L2M displays an energy density of around 20 Wh/kg at a power density of 1800 W/kg. The dramatic increase in the energy density is observed at elevated temperatures: at 60 °C and using an IL-based electrolyte, the energy density reaches 280 Wh/kg at a power density of 11,000 W/kg. To the best of our knowledge, this is the first report on the utilization of L2M as a supercapacitor electrode material at elevated temperatures stemming from its capability to operate by a hybrid storage mechanism in a wide operating voltage window in IL electrolytes.

## Figures and Tables

**Table 1:** Lattice parameters for L2M and L2M2 obtained by the freeze-drying method, followed by annealing at 500 °C.

Formula Unit	(L2M)		(L2M2)	
Space group	*P* 2_1/_*c*		*Pbca*	
Cell parameters				
*a*/Å	4.9933	4.9811 *	8.6979	8.6862 *
*b*/Å	8.3412	8.3140 *	8.8124	8.7922 *
*c*/Å	8.8586	8.8382 *	24.1623	24.1464 *
α/°	90.00	90.00 *	90.00	90.00 *
β/°	121.22	121.25 *	90.00	90.00 *
γ/°	90.00	90.00 *	90.00	90.00 *
V/Å^3^	315.52	312.91 *	1854.33	1844 *

* The data are from references [21,45].

**Table 2:** Infrared bands for ν_3_, ν_1_, ν_4_, and ν_2_ modes of SO_4_^2−^.

	L2M		L2M2	
Vibrational Mode	IR Spectra	Raman Spectra	IR Spectra	Raman Spectra
ν_1_	1018	1024	1033, 1023 980	1034, ~1020 sh 999
ν_2_	491, 467	471, 430	481, 472 451	478, 457 430
ν_3_	1171, 1126 1110	1108, 1094 1085	1178, 1144 1131, 1082 1058	1160, 1135 1118, 1099 1079
ν_4_	640, 616	657, 640 612	667, 653 630, 606	678, 647 623, 606

## Data Availability

Not applicable.
